# Supplementary material for: CRTH2 promotes endoplasmic reticulum stress‐induced cardiomyocyte apoptosis through m‐calpain
Source: EMBO Mol Med. 2018 Jan 15;10(3):e8237. doi: 10.15252/emmm.201708237 (PMC5840549; doi:10.15252/emmm.201708237)
Supplement: Supplementary file 1 — Appendix [file EMMM-10-e8237-s001.pdf]

Supporting Information:

Table of contents

Appendix Table S1. siRNA sequences for adenoviruses construction.

Appendix Table S2. Primers for real-time PCR analysis.

Appendix Figure S1. PG production and PG receptor expression in cardiomyocytes in response to anoxia.

Appendix Figure S2. CRTH2 deletion does not affect necrosis and autophagy in ischemic hearts.

Appendix Figure S3. CRTH2 deficiency has no overt influence on myocardial angiogenesis in mice after MI.

Appendix Figure S4. Loss of CRTH2 does not affect the recruitment of macrophages and neutrophils to the infarcted hearts in mice after MI.

Appendix Figure S5. CRTH2 deficiency retards T cell infiltration in the peri-infarct zones of MI mouse heart.

Appendix Figure S6. CRTH2 deficiency has not evident effect on mitochondrial and death receptor-mediated myocardial apoptosis in mice after MI.

Appendix Figure S7. CRTH2 activation facilitates ER stress-induced cardiomyocyte apoptosis by promoting caspase-12-dependent apoptotic pathway.

Appendix Figure Legends.

Appendix Tables

Table S1

siRNA sequences for adenoviruses construction

siRNA	Sense	Antisense
capase-12	GCTCTCATCATCTGCAACAAA	TTTGTTGCAGATGATGAGAGC
μ-calpain	GCCGTGGACTTTGACAACTTT	AAAGTTGTCAAAGTCCACGGC
m-calpain	GCGGTCAGATACCTTCATTAA	TTAATGAAGGTATCTGACCGC
calpain-7	CCTCATTACTTGACTAAGATA	TATCTTAGTCAAGTAATGAGG
Human caspase-4	GACAGCACAATGGGCTCTATC	GATAGAGCCCATTGTGCTGTC

Table S2

Primers for real-time PCR analysis

Gene	Forward	Reverse
DP1	AACCTCTATGACATGCACAGGCG	AAGGCTTGGAGGTCTTCTGAGTC
CRTH2	AGATGGTCCAGCTTCCAAACC	ACAGGATGAGTCCGTTTTCCA
TNFα	CCCTCACACTCAGATCATCTTCT	GCTACGACGTGGGCTACAG
IL-1β	GCAACTGTTCCTGAACTCAACT	ATCTTTTGGGTCCGTCAACT
IL-12a	ATGGCCATGTGGGAGCTGGAGAAAG	GTGGAGCAGCAGATGTGAGTGGCT
IL-18	GACTCTTGCGTCAACTTCAAGG	CAGGCTGTCTTTTGTCAACGA
IL-10	GCTCTTACTGACTGGCATGAG	CGCAGCTCTAGGAGCATGTG
TGF-β	CCGCAACAACGCCATCTATG	CCCGAATGTCTGACGTATTGAAG
IL-4	GGTCTCAACCCCCAGCTAGT	GCCGATGATCTCTCTCAAGTGAT
IL-5	CTCTGTTGACAAGCAATGAGACG	TCTTCAGTATGTCTAGCCCCTG
IL-13	CCTGGCTCTTGCTTGCCTT	GGTCTTGTGTGATGTTGCTCA
IFN-γ	ATGAACGCTACACACTGCATC	CCATCCTTTTGCCAGTTCCTC
VEGF	GCACATAGAGAGAATGAGCTTCC	CTCCGCTCTGAACAAGGCT
FGF	GCGACCCACACGTCAAACTA	CCGTCCATCTTCCTTCATAGC
HGF	ATGTGGGGGACCAAACTTCTG	GGATGGCGACATGAAGCAG
PDGF	GAGGAAGCCGAGATACCCC	TGCTGTGGATCTGACTTCGAG
Actc1	GTGCCAGGATGTGTGACGA	CTGTCCCATACCCACCATGAC
cTNT	CAGAGGAGGCCAACGTAGAAG	CTCCATCGGGGATCTTGGGT
Myh6	GCCCAGTACCTCCGAAAGTC	GCCTTAACATACTCCTCCTTGTC
Myl7	GGCACAACGTGGCTCTTCTAA	TGCAGATGATCCCATCCCTGT
Ryr2	ATGGCTTTAAGGCACAGCG	CAGAGCCCGAATCATCCAGC
Scn5a	ATGGCAAACTTCCTGTTACCTC	CCACGGGCTTGTTTTTCAGC
Slc8a1	CGCTGGGGAAGATGACGATG	TGGGACGAAGGCAAACAGAAC
Gja1	ACAGCGGTTGAGTCAGCTTG	GAGAGATGGGGAAGGACTTGT
Atp2a2	GAGAACGCTCACACAAAGACC	CAATTCGTTGGAGCCCCAT
Cacna1c	ATGAAAACACGAGGATGTACGTT	ACTGACGGTAGAGATGGTTGC
Kcna5	TCCGACGGCTGGACTCAATAA	CAGATGGCCTTCTAGGCTGTG
Pln	AAAGTGCAATACCTCACTCGC	GGCATTTCAATAGTGGAGGCTC
Bax	AGACAGGGGCCTTTTTGCTAC	AATTCGCCGGAGACACTCG
Bad	CTCCGAAGGATGAGCGATGAG	TTGTCGCATCTGTGTTGCAGT
Bak	GACTTTTGGCGTCATCTCATCG	TTCATCCCAATCAGAACAGCC
Bid	CCAGTCACGCACCATCTTTG	GTCCATCTCGTTTCTAACCAAGT
Bim	CCCGGAGATACGGATTGCAC	GCCTCGCGGTAATCATTTGC
Bcl-2	GCTACCGTCGTGACTTCGC	CCCCACCGAACTCAAAGAAGG
Bcl-xl	GACAAGGAGATGCAGGTATTGG	TCCCGTAGAGATCCACAAAAGT
Bcl-w	GCGGAGTTCACAGCTCTATAC	AAAAGGCCCCTACAGTTACCA
Caspase-12	TAGGGGAAAGTGCGAGTTTCA	GGGCCAATCCAGCATTTACCT
μ-Calpain	CTCCGGTTTCTCATAGCCTGG	GCAGAATAGTCTCGTTGAGGGTG

Table S2 continued

Gene	Forward	Reverse
m-Calpain	CTCGGGGCATCGAATGGAAG	TGCCTGCATAGTTTTCCTGGA
Calpain 3	GTGGTCATTGACGACTGTCTG	CACTCGGAGCATCCTTGATCT
Calpain 5	GATCCCCGGCTCTTCGTAGAT	GACGATTACGTCCACCCACTC
Calpain 6	GGAAGCGTCCACAGGACATTT	GTGGTTGGGAATTGCCTTTGT
Calpain7	GAAGGCCGCTACTCTGAGG	GGGCTTGCACTCTTTCCAGAT
Calpain 8	AGAACTACCCAGCCACTTACTG	GCTCATCGGTGTGATTCTCCA
Calpain 9	AAACGGCCAGGGGAAATTGT	ATATCCCGGCATAGCCAGAAC
Calpain 10	CTGCGGATTCACAACCCCT	CCTCGACCCAGAACTCTCCTT
GAPDH	CCCTTATTGACCTCAACTACATGGT	GAGGGGCCATCCACAGTCTTCTG
Human caspase-4	CAAGAGAAGCAACGTATGGCA	AGGCAGATGGTCAAACTCTGTA
Human GAPDH	CTGGGCTACACTGAGCACC	AAGTGGTCGTTGAGGGCAATG

Appendix Figure S1

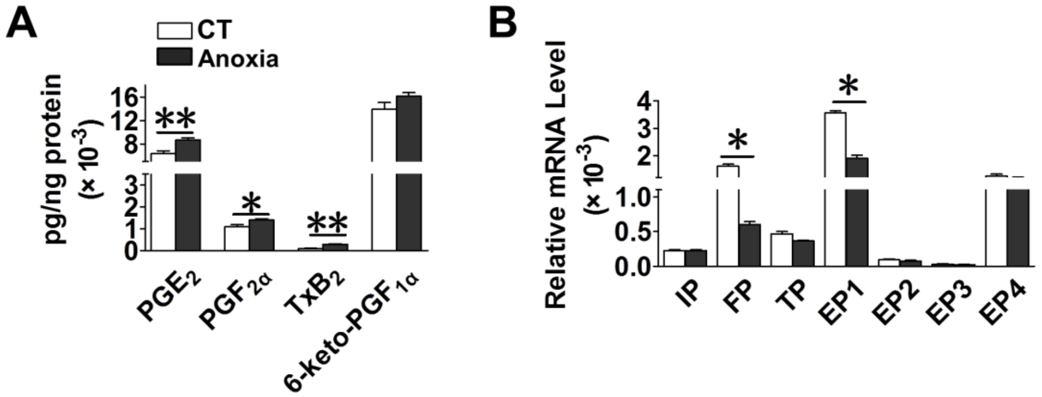

Appendix Figure S2

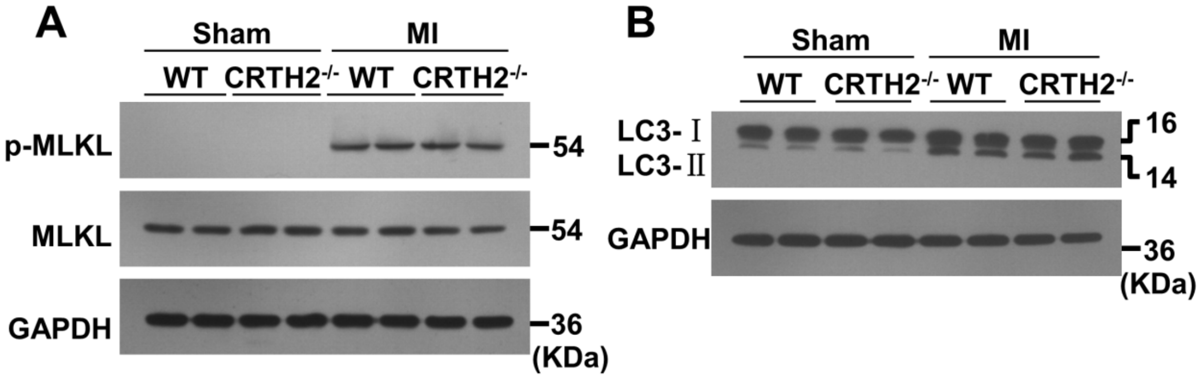

Appendix Figure S3

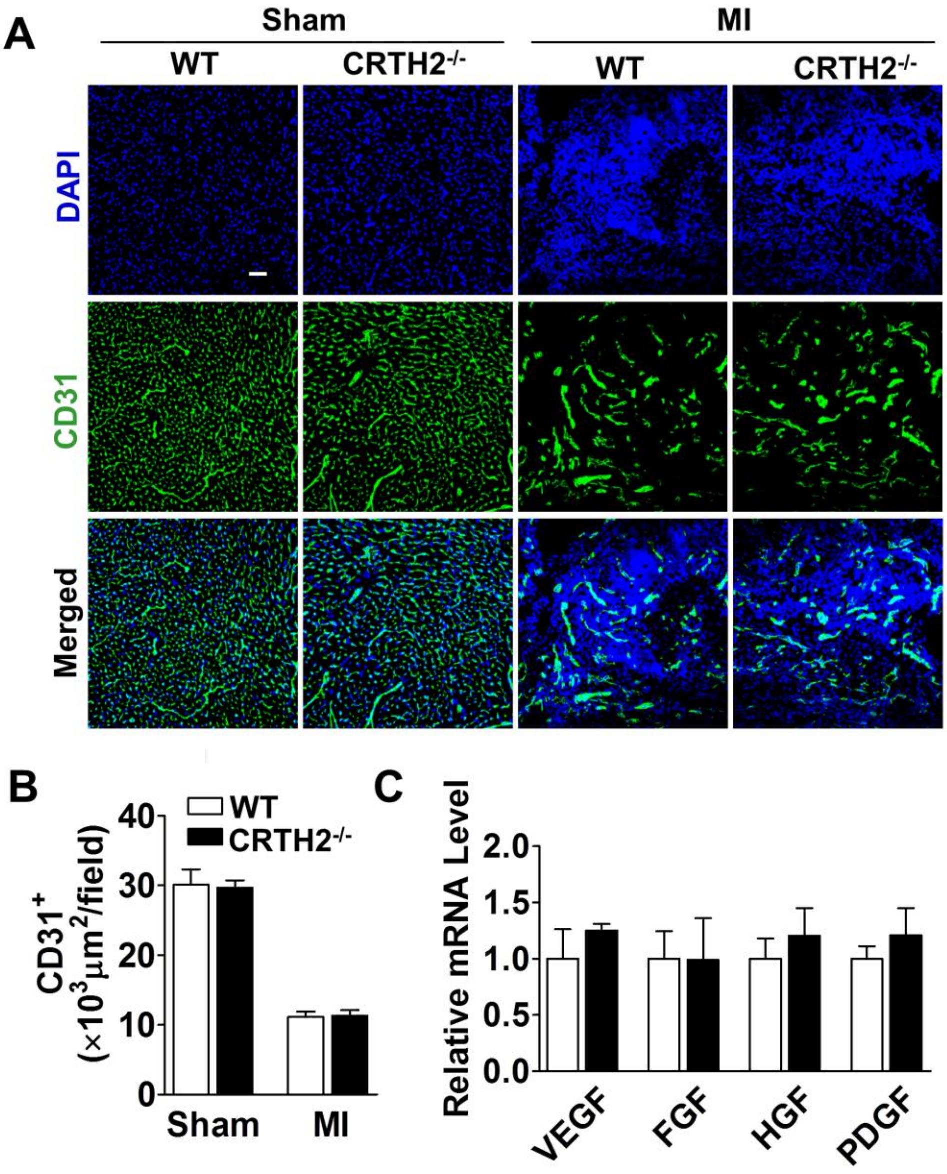

Appendix Figure S4

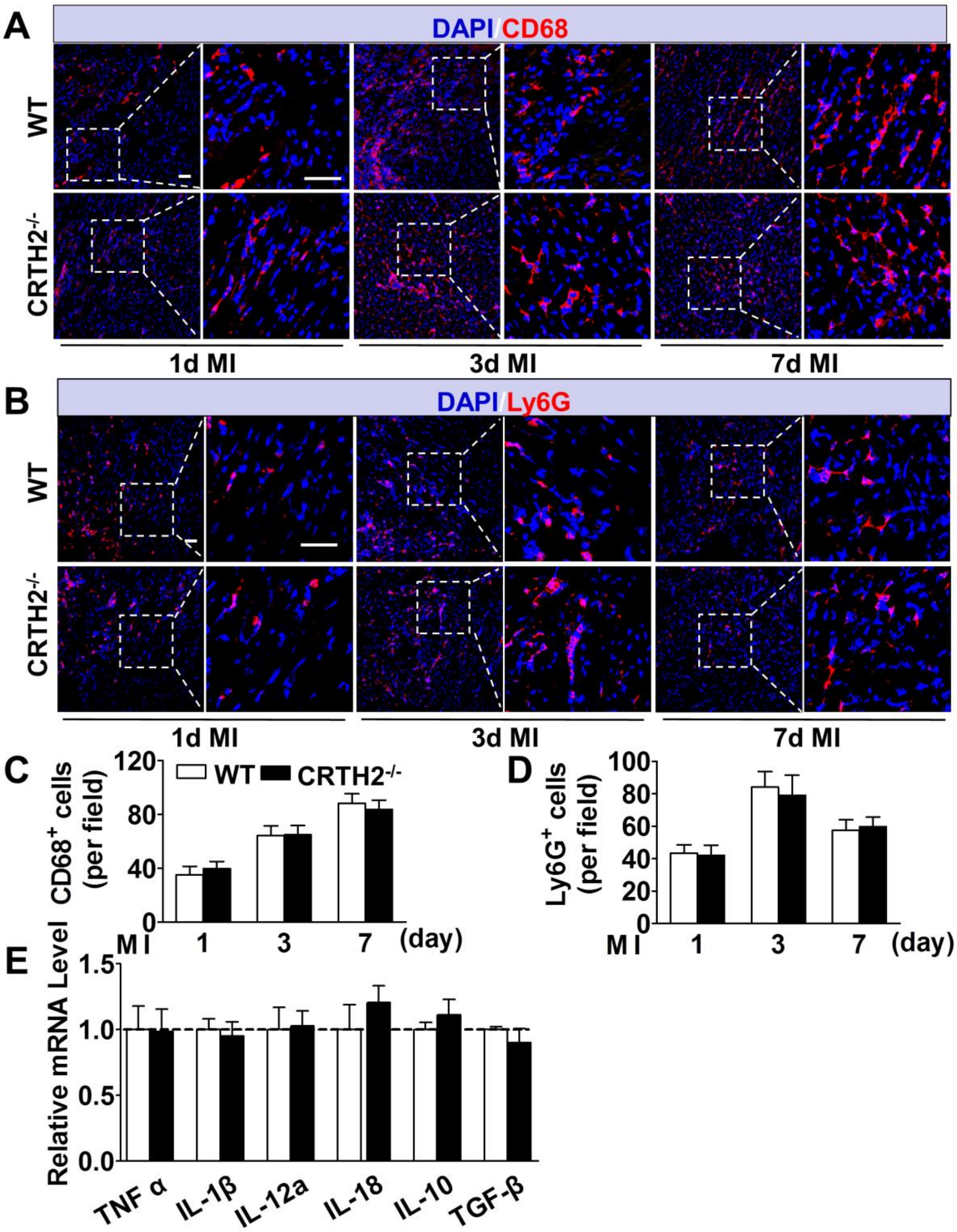

Appendix Figure S5

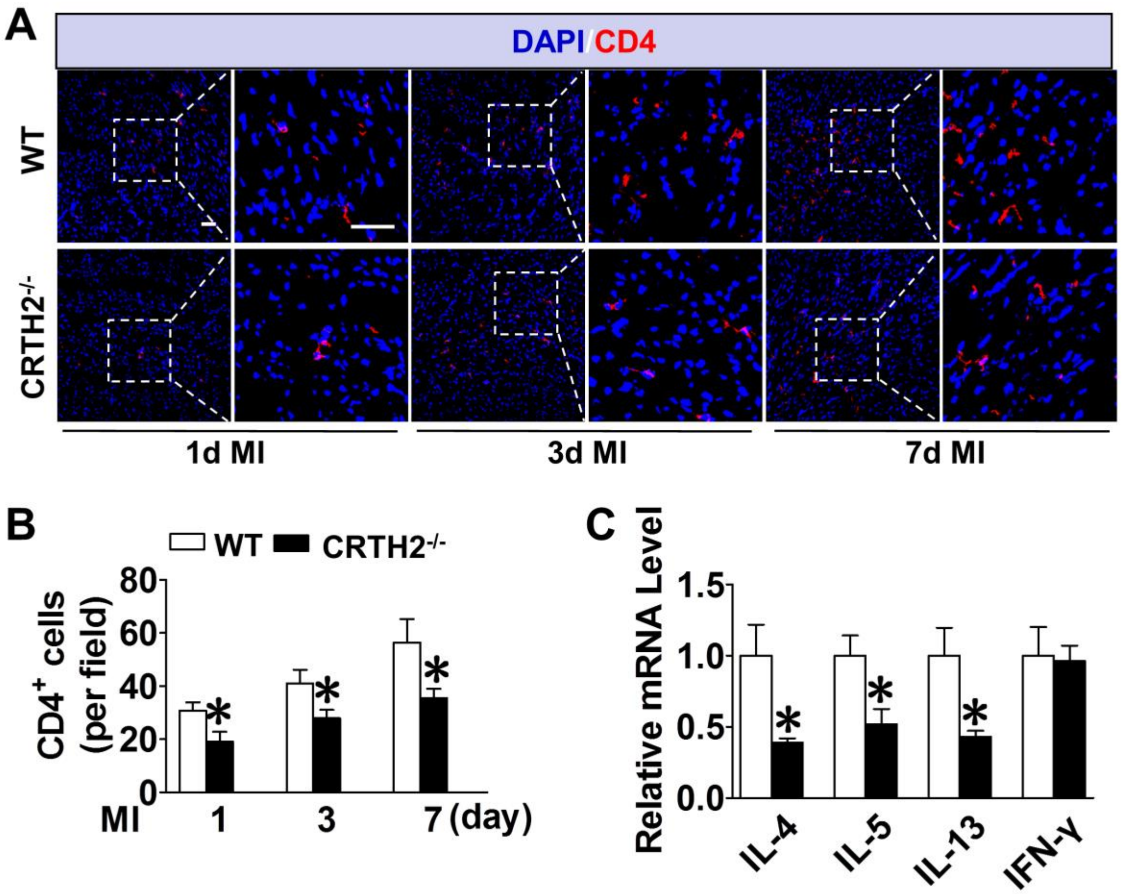

Appendix Figure S6

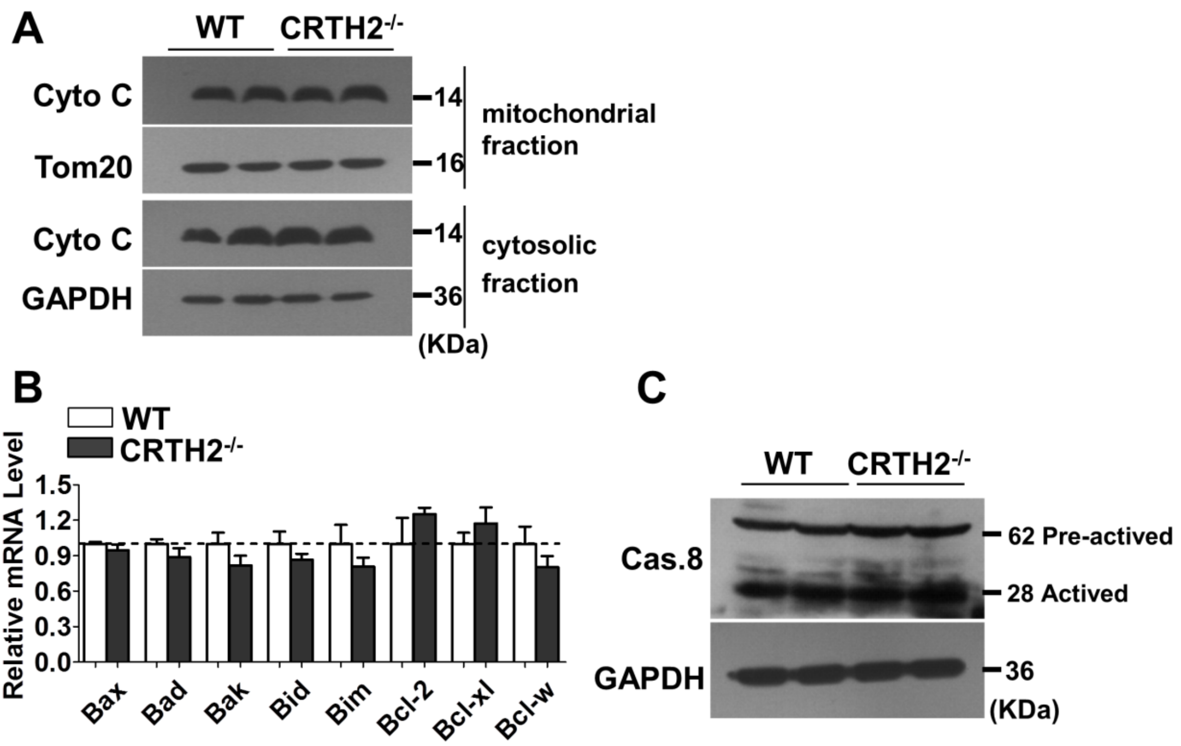

Appendix Figure S7

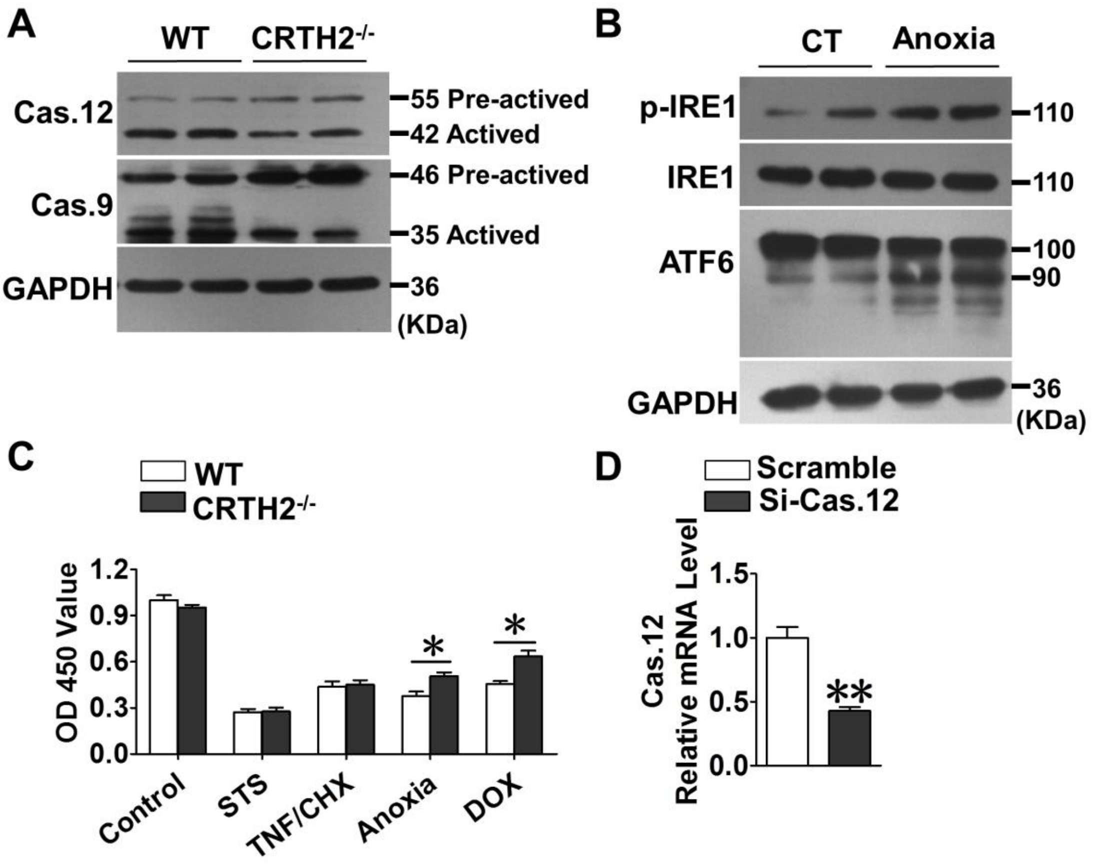

Appendix figure legends

Figure S1. PG production and PG receptor expression in cardiomyocytes in response to anoxia.

A, The PG profiles of neonatal mouse cardiomyocytes challenged by anoxia for 1 h. Data represent mean ± SEM. PGE_2, ** P = 0.00327, vs control, $PGF_{2\alpha}$,*P = 0.021, vs control, TxB_2, ** P<0.0001, vs control (Mann-Whitney U test); n = 6.

B, Relative mRNA levels of PG receptors in mouse cardiomyocytes exposed to anoxia. Data represent mean ± SEM. FP, *P<0.0001, vs control, EP1, *P< 0.0001, vs control (Mann-Whitney U test); n = 5.

Figure S2. CRTH2 deletion does not affect necrosis and autophagy in ischemic hearts.

A,Western blot analysis of phosphorylation of MLKL in the infarcted mouse heart 24 hours post-MI.

B,Western blot analysis of LC3 in the infarcted mouse heart 24 hours post-MI.

Figure S3. CRTH2 deficiency has no overt influence on myocardial angiogenesis in mice after MI.

A, Representative images of immunofluorescence staining of CD31 in the peri-infarct region of mouse heart at day 14 after MI. Green, $CD31^+$ cells; blue, DAPI-stained nuclei. Scale bar, 50 μm.

B, Quantitation of the $CD31^+$ cells in (A). Data represent mean ± SEM; WT and $CRTH2^{-/-}$ (Sham), n = 5; WT and $CRTH2^{-/-}$(MI), n = 8.

C, Angiogenesis-related genes expression profile in the infarct border zone of mouse heart at 14 days after MI. Data represent mean ± SEM; n = 5.

Figure S4. Loss of CRTH2 does not affect the recruitment of macrophages and neutrophils to the infarcted hearts in mice after MI.

A, Representative images of distribution of macrophages ($CD68^+$) in the infarct border zone of mouse heart at days 1, 3, and 7 after MI. Blue, DAPI-stained nuclei; red, CD68-positive cell; scale bar, 50 μm.

B, Representative images of distribution of neutrophils ($Ly6G^+$) in the infarct border zone of mouse at days 1, 3, and 7 after MI. Blue, DAPI-stained nuclei; red, Ly6G-positive cell; scale bar, 50 μm.

C, Quantification of total $CD68^+$ cells in (A).

D, Quantification of total Ly6G$^+$ cells in (B). Data represent mean ± SEM; *n* = 7.

E, Relative mRNA levels of inflammatory cytokines mainly produced by macrophages and neutrophils in the infarcted mouse heart at day 3 post MI. Data represent mean ± SEM; *n* = 5.

Figure S5. CRTH2 deficiency retards T cell infiltration in the peri-infarct zones of MI mouse heart.

A, Representative images of distribution of T cells (CD4$^+$) in the infarct border zone of mouse heart at days 1, 3, and 7 after MI. Blue, DAPI-stained nuclei; red, CD4-positive cell; scale bar, 50 μm.

B, Quantification of total CD4$^+$ cells in (A). Data represent mean ± SEM. MI day 1, **P* =0.0306,vs WT; MI day 3, **P* = 0.0481, vs WT; MI day 7, **P* = 0.0454, vs WT (unpaired two-tailed t test); *n* = 8.

C, Relative mRNA levels of inflammatory cytokines mainly produced by T cells in the infarcted mouse heart at day 3 post MI. Data represent mean ± SEM. IL4, **P* =0.0242,vs WT; IL5, **P* = 0.0267, vs WT; IL13, **P* = 0.0214, vs WT (unpaired two-tailed t test); *n* = 5.

Figure S6. CRTH2 deficiency has not evident effect on mitochondrial and death receptor-mediated myocardial apoptosis in mice after MI.

A, Western blot analysis of the release of cytochrome C in cardiac tissues at border zones in mice after MI.

B, Relative mRNA levels of mitochondrial apoptosis-related genes in cardiac tissues at border zones of MI mouse heart. Data represent mean ± SEM; WT, *n* = 4, CRTH2$^{-/-}$, *n* = 6.

C, Western blot analysis of caspase-8 in cardiac tissues at border zones of MI mouse heart.

Figure S7. CRTH2 activation facilitates ER stress-induced cardiomyocyte apoptosis by promoting caspase-12-dependent apoptotic pathway.

A, Western blot analysis of caspase-12 and caspase-9 activity in cardiac tissues at border zones of MI mouse heart.

B, Western blot analysis of ER stress markers p-IRE1 and cleaved ATF6 in mouse cardiomyocytes in response to anoxia.

C, Survival of cardiomyocytes in response to ER stress- and non-ER stress-induced apoptotic stimuli was assessed using CCK-8 assays. STS, staurosporine (2 mM); TNF-α/CHX, TNF-α (100 ng/mL)

plus cycloheximide (10 mg/mL); DOX, doxorubicin. Data represent mean ± SEM. Anoxia, *P =0.00541, vs WT; DOX, *P = 0.000944, vs WT, (Mann-Whitney U test); n = 8.

D, Quantitative real-time polymerase chain reaction (qRT-PCR) depicted the efficiency of adenovirus-mediated siRNA targeting caspase-12 (Si-Cas.12) in mouse cardiomyocytes. Data represent mean ± SEM. **P < 0.0001, vs scrambled siRNA (Mann-Whitney U test); n = 6.
