# Supplementary material for: CRTH2 promotes endoplasmic reticulum stress‐induced cardiomyocyte apoptosis through m‐calpain
Source: EMBO Mol Med. 2018 Jan 15;10(3):e8237. doi: 10.15252/emmm.201708237 (PMC5840549; doi:10.15252/emmm.201708237)
Supplement: Supplementary file 2 — Expanded View Figures PDF [file EMMM-10-e8237-s002.pdf]

Expanded View Figures

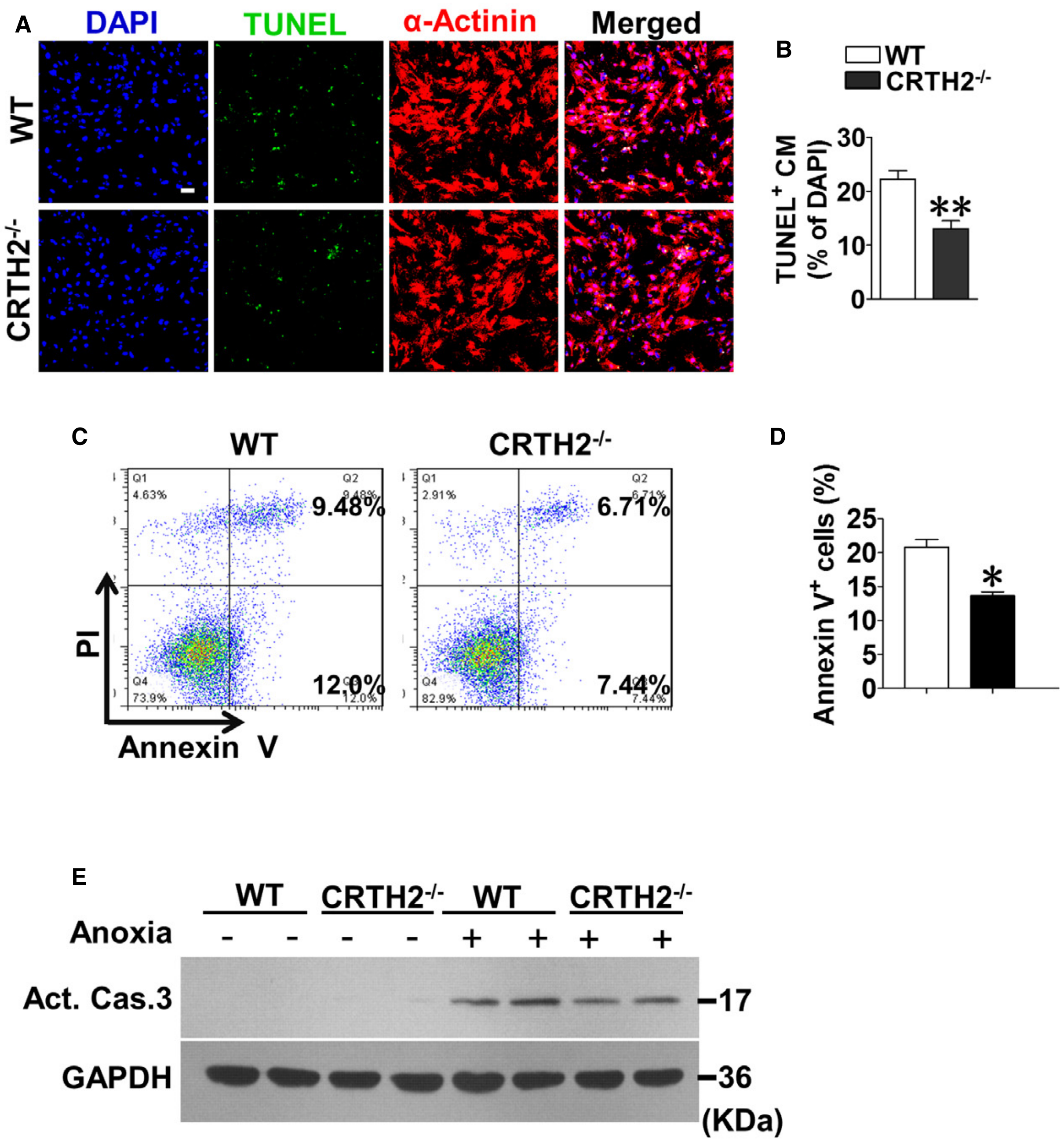

Figure EV1. CRTH2 deletion attenuates anoxia-induced cardiomyocyte apoptosis *in vitro*.

- A Representative TUNEL-stained images of mouse cardiomyocytes challenged with anoxia. Green, TUNEL-positive nuclei; blue, DAPI-stained nuclei; red, cardiomyocytes labeled with antibody to α-actinin; scale bar, 50 μm.
- B Quantification of TUNEL-positive cells in (A). Data represent mean ± SEM. $^{**}P$ = 0.00202, vs. WT (Mann–Whitney U-test); n = 6.
- C Flow cytometry analysis of annexin V and propidium iodide (PI) staining of apoptotic cardiomyocytes following anoxia treatment.
- D Quantification of annexin V^+ cells in (C). Data represent mean ± SEM. $^{*}P$ = 0.0002, vs. WT (unpaired two-tailed t-test); n = 6.
- E Western blot analysis of activated caspase-3 in mouse cardiomyocytes challenged with anoxia.

Source data are available online for this figure.

© 2018 The Authors

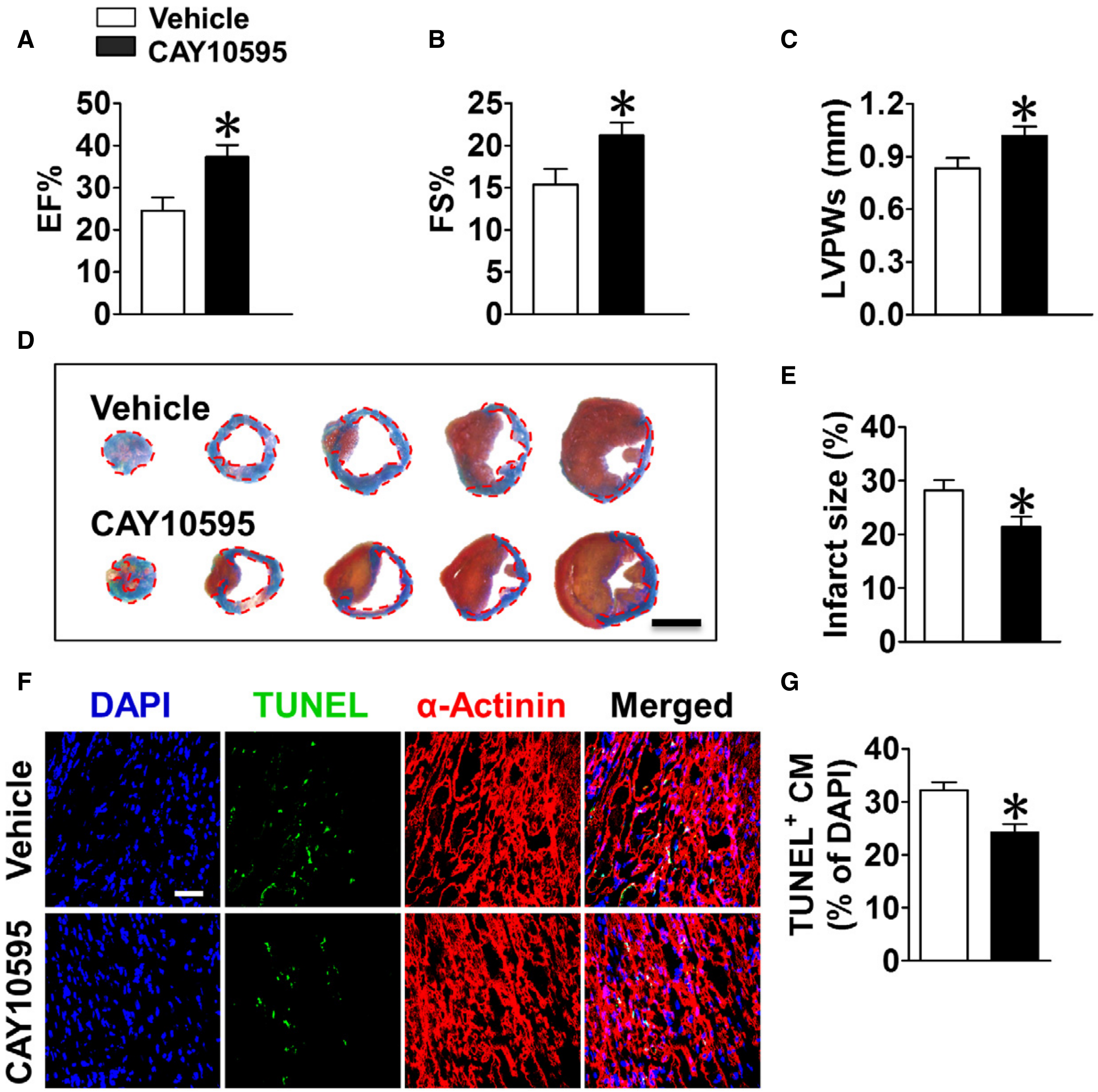

Figure EV2. CRTH2 inhibition with selective antagonist CAY10595 protects the hearts from MI in mice.

A–C Echocardiographic analysis of cardiac function in mice with or without CAY10595 (5 mg/kg/day) treatment at day 14 post-MI. EF, ejection fraction (A); FS, fractional shortening (B); LVPWs, systolic left ventricle posterior wall thickness at end-systole (C). Data represent mean ± SEM. EF, *P = 0.0346, vs. Vehicle; FS, *P = 0.0138, vs. Vehicle; LVPWs, *P = 0.0129, vs. Vehicle (unpaired two-tailed *t*-test); Vehicle, n = 8; CAY10595, n = 12.

D Representative images of Evans blue and TTC staining of infarcted mouse heart at day 14 post-MI. Scale bar, 500 μm.

E Quantification of infarcted size of mouse heart in (D). Data represent mean ± SEM. *P = 0.0172, vs. vehicle (unpaired two-tailed *t*-test); Vehicle, n = 8; CAY10595, n = 10.

F Representative TUNEL-stained images of the peri-infarct region in MI mouse heart. Green, TUNEL-positive nuclei; blue, DAPI-stained nuclei; red, cardiomyocytes labeled with antibody to α-actinin; scale bar, 50 μm.

G Quantification of TUNEL-positive cardiomyocytes in (F). Data represent mean ± SEM. *P = 0.0163, vs. vehicle (unpaired two-tailed *t*-test); n = 12.

© 2018 The Authors

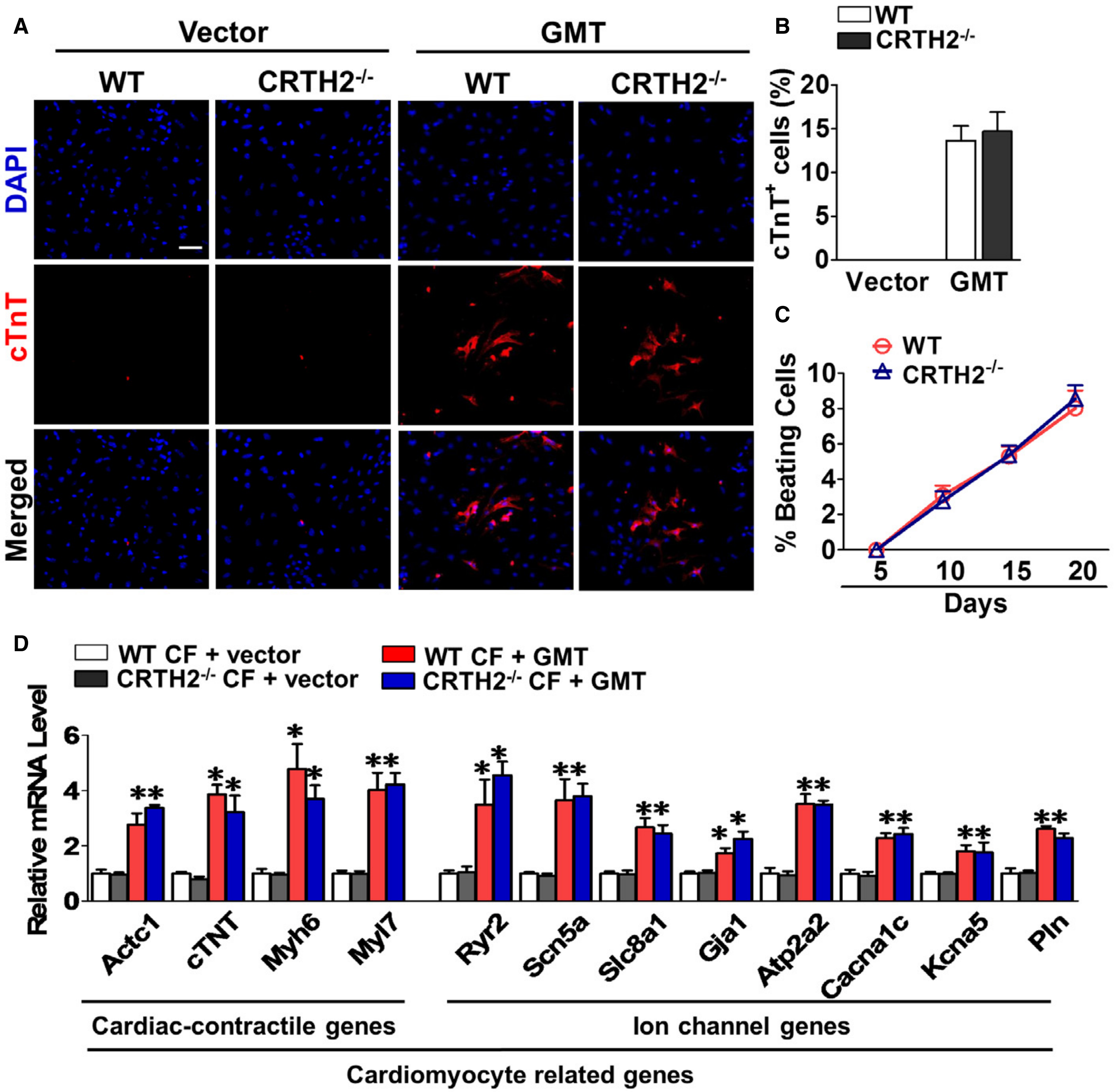

Figure EV3. Effect of CRTH2 deletion on reprogramming of cardiac fibroblasts into cardiomyocytes.

A Representative images of immunofluorescence staining of cardiac troponin T (cTnT) in cardiac fibroblasts (CFs) after co-transfection of three transcription factors—Gata4, Mef2c, and Tbx5 (GMT). Blue, DAPI-stained nuclei; red, cTnT-positive cells; scale bar, 50 μm.

B Quantification of total $cTnT^+$ cells in (A). Data represent mean ± SEM; WT and $CRTH2^{-/-}$ (Vector), n = 6; WT and $CRTH2^{-/-}$ (MI), n = 8.

C Percentage of beating cells among cTnT-positive cells. Data represent mean ± SEM; n = 5.

D Cardiac-specific gene expression in GMT-infected CFs at 2 weeks postinfection. Data represent mean ± SEM. *P < 0.05, vs. vector (unpaired two-tailed t-test); n = 6.

 © 2018 The Authors

Figure EV4. CRTH2$^{+/+}$ bone marrow reconstitution does not influence cardiac repair after MI in CRTH2$^{-/-}$ mice.

A Genotyping of the respective transplanted BM in mice.

B Representative images of recruitment of CD4^{+} cells in the infarct border zone of mouse heart undergoing BM transplantation at day 7 after MI. Blue, DAPI-stained nuclei; red, CD4-positive cell; scale bar, 50 μm.

C Quantification of total CD4^{+} cells in (B). Data represent mean ± SEM. *P = 0.0125, KO→WT vs. WT→WT; *P = 0.0148, KO→KO vs. WT→KO (unpaired two-tailed *t*-test); n = 7.

D Representative TUNEL-stained images of the peri-infarct zone of mouse heart undergoing BM transplantation at day 1 after MI. Green, TUNEL-positive nuclei; blue, DAPI-stained nuclei; red, cardiomyocytes labeled with antibody to α-actinin; scale bar, 50 μm.

E Quantification of TUNEL-positive cells in (D). Data represent mean ± SEM. P = 0.606, KO→WT vs. WT→WT; P = 0.631, KO→KO vs. WT→KO (unpaired two-tailed *t*-test); n = 9.

F, G M-mode echocardiographic analysis of cardiac function in BM transplanted mice at day 14 after MI. EF, ejection fraction (F); FS, fractional shortening (G). Data represent mean ± SEM. EF, P = 0.371, KO→WT vs. WT→WT; P = 0.896, KO→KO vs. WT→KO; FS, P = 0.726, KO→WT vs. WT→WT; P = 0.678, KO→KO vs. WT→KO (unpaired two-tailed *t*-test); WT→WT, n = 8, KO→WT, n = 8, WT→KO, n = 8, KO→KO, n = 6.

Source data are available online for this figure.

© 2018 The Authors

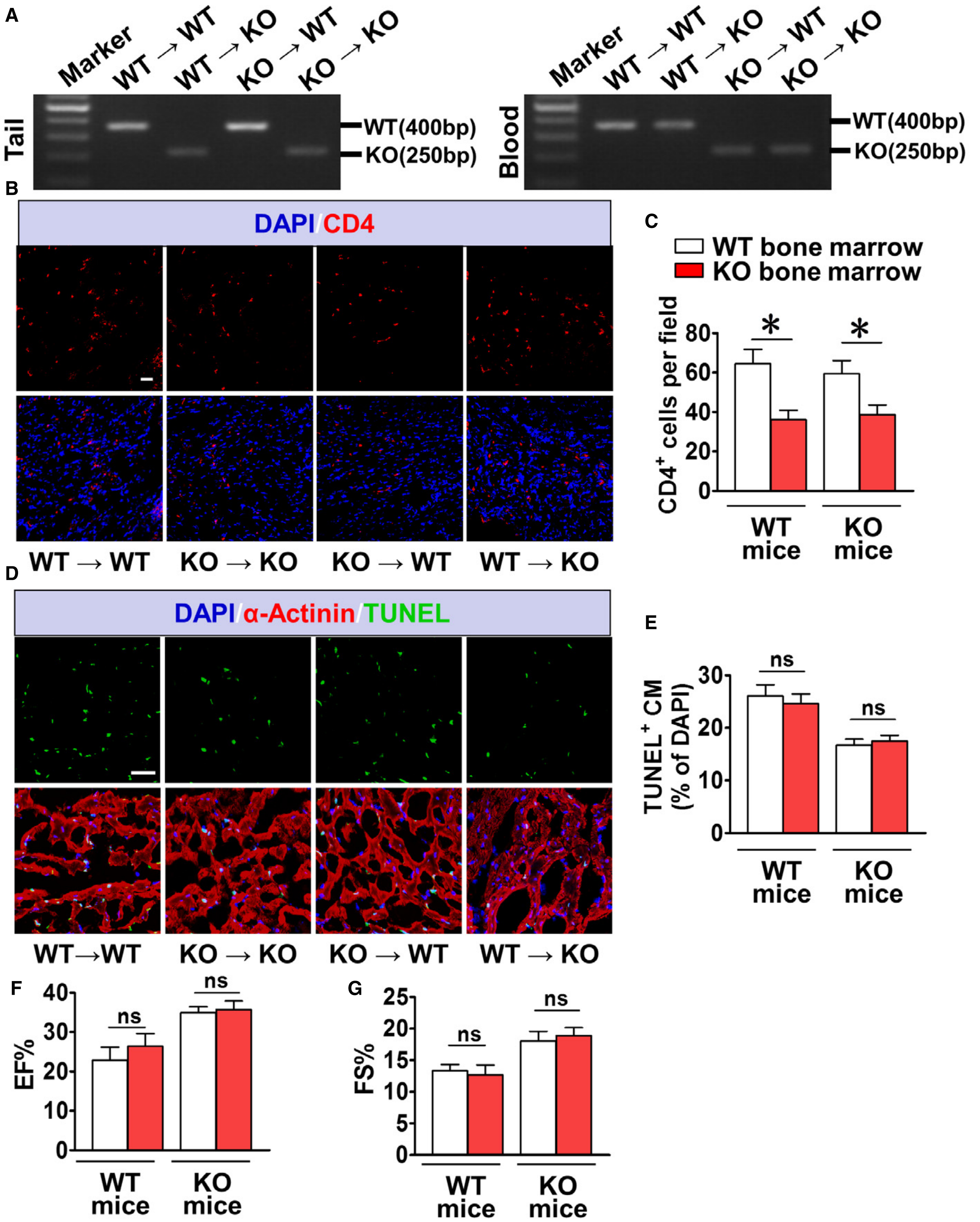

Figure EV4.

© 2018 The Authors

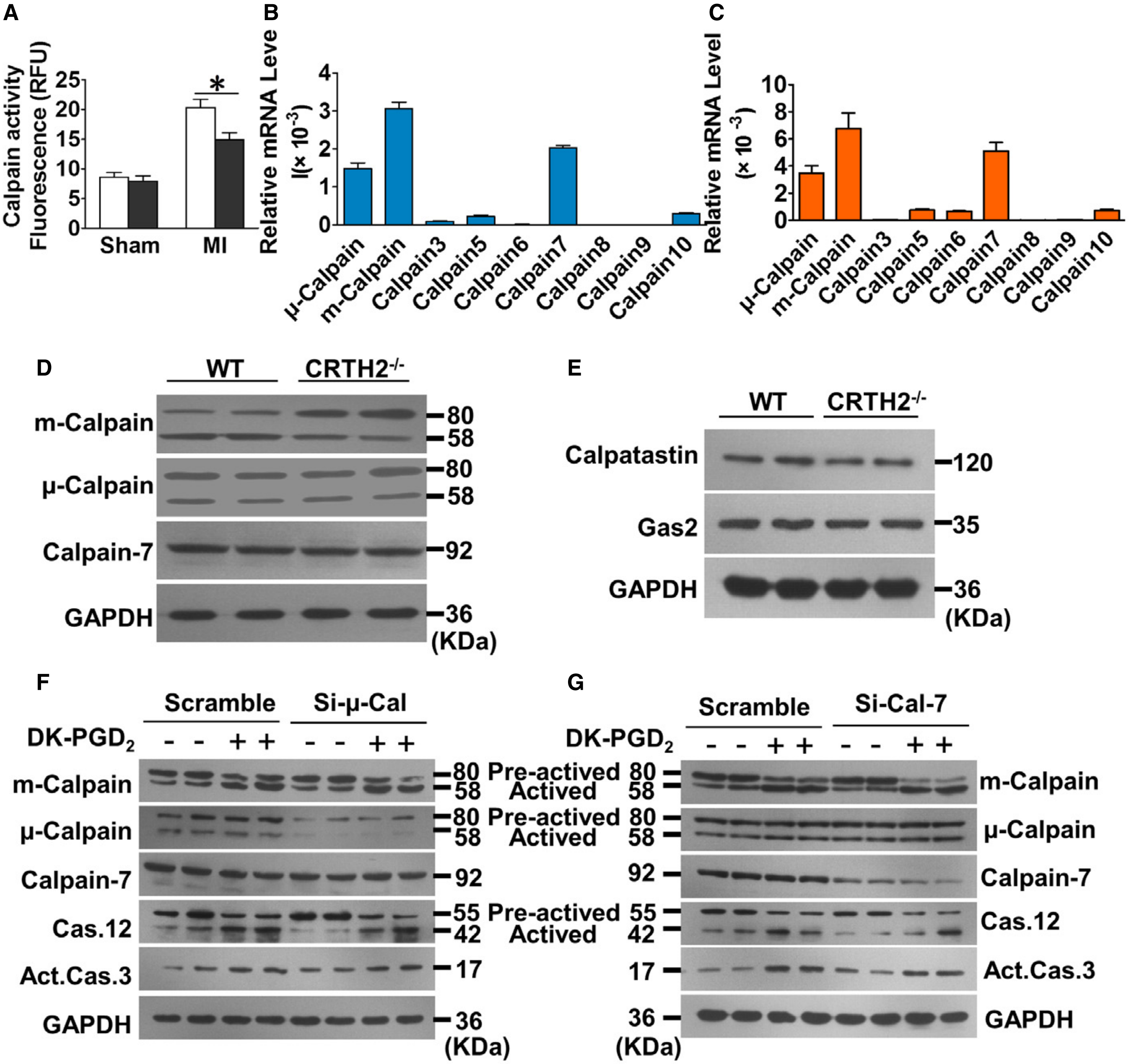

Figure EV5. CRTH2 mediates ER stress-induced cardiomyocyte apoptosis independent of μ-calpain and calpain-7.

A Calpain activity in the border zones of infarcted hearts. Data represent mean ± SEM. *P = 0.0132, vs. WT (Mann–Whitney U-test); WT and CRTH2$^{-/-}$ (Sham), n = 5; WT and CRTH2$^{-/-}$ (MI), n = 6.

B, C Relative mRNA levels of calpain isoforms in mouse cardiomyocytes (B) and in the border zones of infarcted hearts (C). Data represent mean ± SEM; n = 6.

D Western blot analysis of m-calpain, μ-calpain, and calpain-7 in cardiac tissues at border zones of mouse heart at 24 h after MI.

E Western blot analysis of the expression of Gas2 and calpastatin in infarcted hearts of MI mice.

F Western blot analysis of calpain and caspase family members in si-μ-Cal-infected mouse cardiomyocytes after treatment of DK-PGD$_2$ under anoxia condition.

G Western blot analysis of calpain and caspase family members in si-Cal-7-infected mouse cardiomyocytes after treatment of DK-PGD$_2$ under anoxia condition.

Source data are available online for this figure.

© 2018 The Authors
