# Supplementary figures and images for: CRTH2 promotes endoplasmic reticulum stress‐induced cardiomyocyte apoptosis through m‐calpain
Source: EMBO Mol Med. 2018 Jan 15;10(3):e8237. doi: 10.15252/emmm.201708237 (PMC5840549; doi:10.15252/emmm.201708237)

Appendix Figure S6A

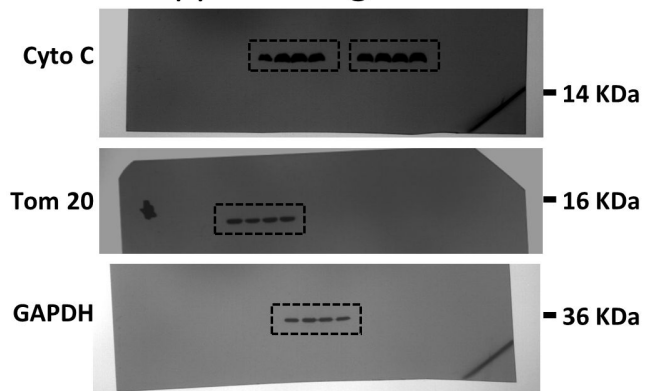

Appendix Figure S6C

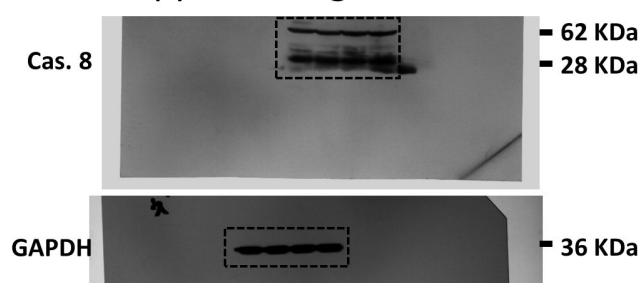

Supplement: Supplementary file 3 — Source Data for Expanded View and Appendix [file EMMM-10-e8237-s011.zip › Appendix_Figure_S6.pdf]

Appendix Figure S7A

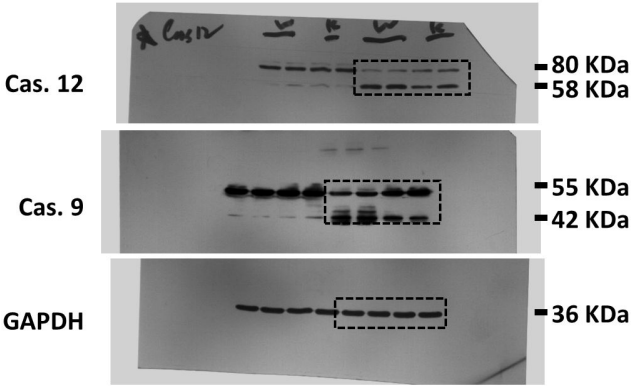

Appendix Figure S7B

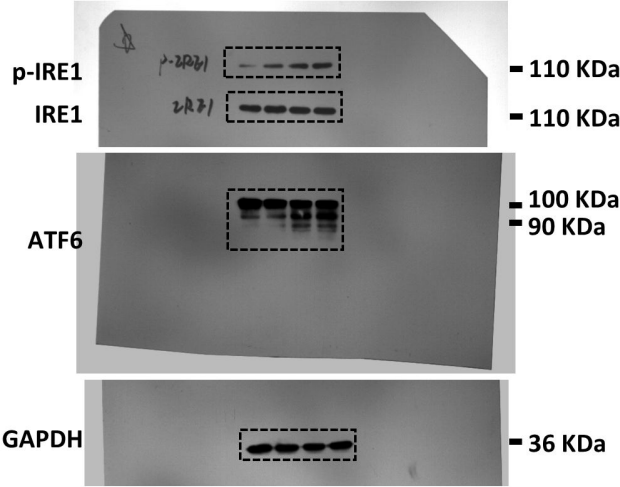

Supplement: Supplementary file 3 — Source Data for Expanded View and Appendix [file EMMM-10-e8237-s011.zip › Appendix_Figure_S7.pdf]

## Expand View Figure 1E

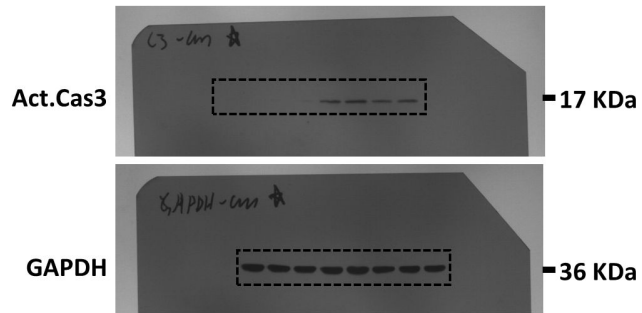

Supplement: Supplementary file 3 — Source Data for Expanded View and Appendix [file EMMM-10-e8237-s011.zip › Expand_View_Figure_1.pdf]

Expand View Figure 4A

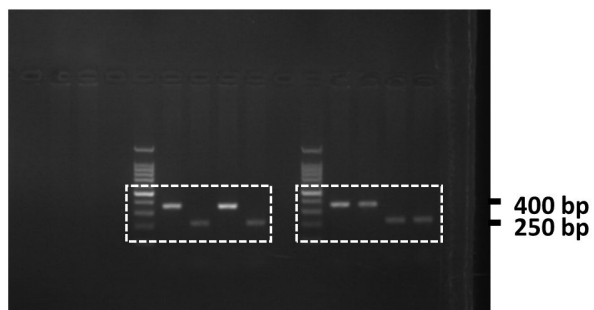

Supplement: Supplementary file 3 — Source Data for Expanded View and Appendix [file EMMM-10-e8237-s011.zip › Expand_View_Figure_4.pdf]

Expand View Figure 5D

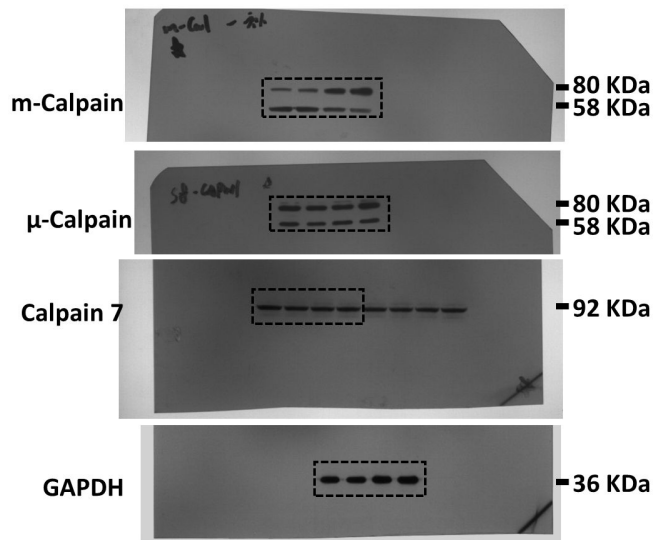

Expand View Figure 5E

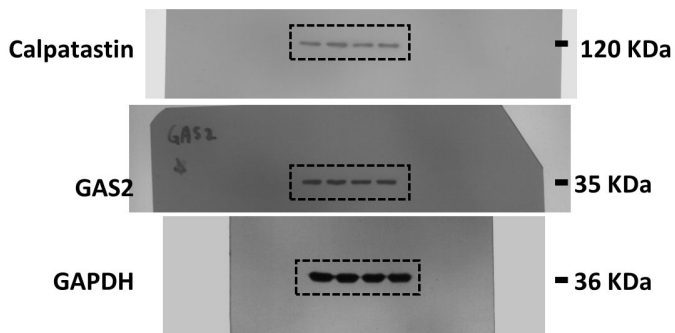

Expand View Figure 5F

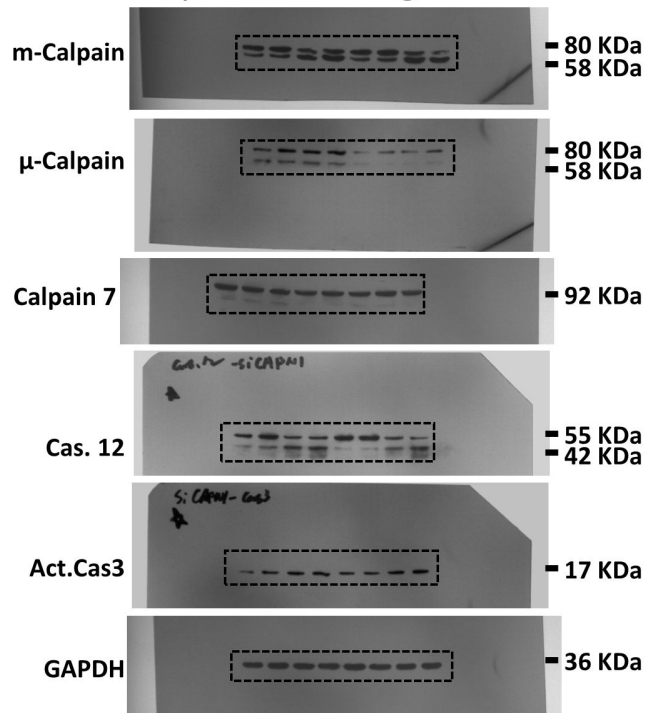

Expand View Figure 5G

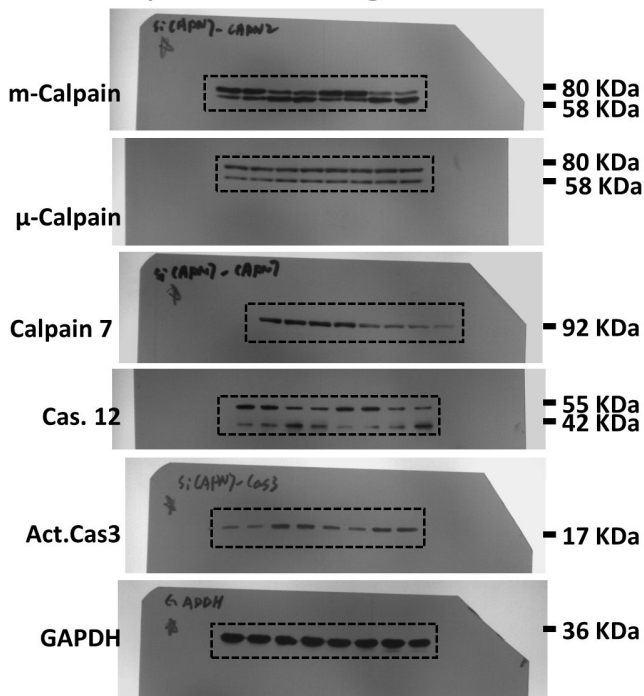

Supplement: Supplementary file 3 — Source Data for Expanded View and Appendix [file EMMM-10-e8237-s011.zip › Expand_View_Figure_5.pdf]

Appendix Figure S2A

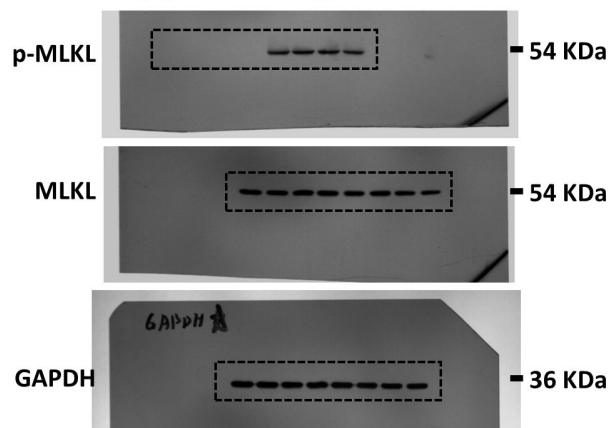

Appendix Figure S2B

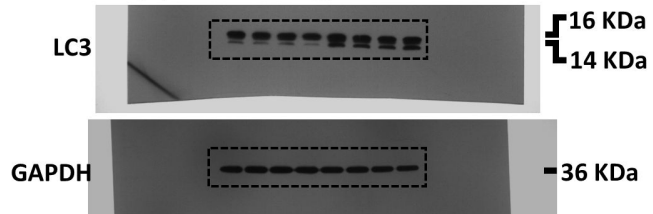

Supplement: Supplementary file 3 — Source Data for Expanded View and Appendix [file EMMM-10-e8237-s011.zip › Appendix_Figure_S2.pdf]

Figure 1C

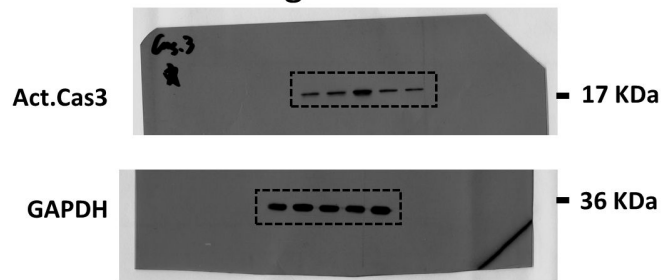

Supplement: Supplementary file 5 — Source Data for Figure 1 [file EMMM-10-e8237-s003.pdf]

Figure 2C

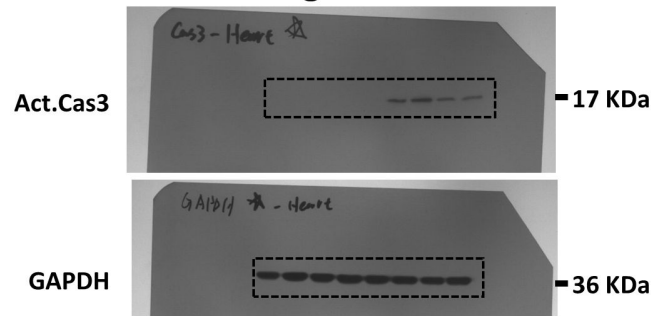

Supplement: Supplementary file 6 — Source Data for Figure 2 [file EMMM-10-e8237-s004.pdf]

Figure 3C

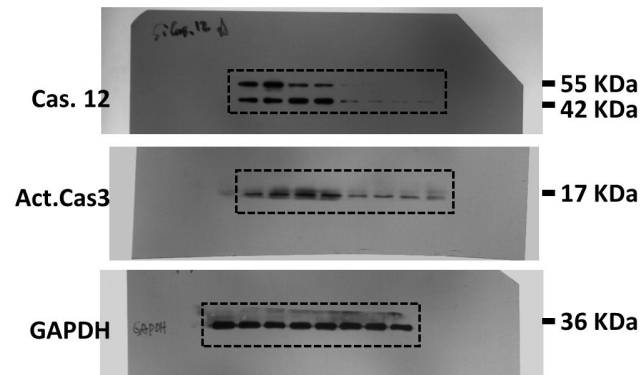

Supplement: Supplementary file 7 — Source Data for Figure 3 [file EMMM-10-e8237-s005.pdf]

Figure 4D

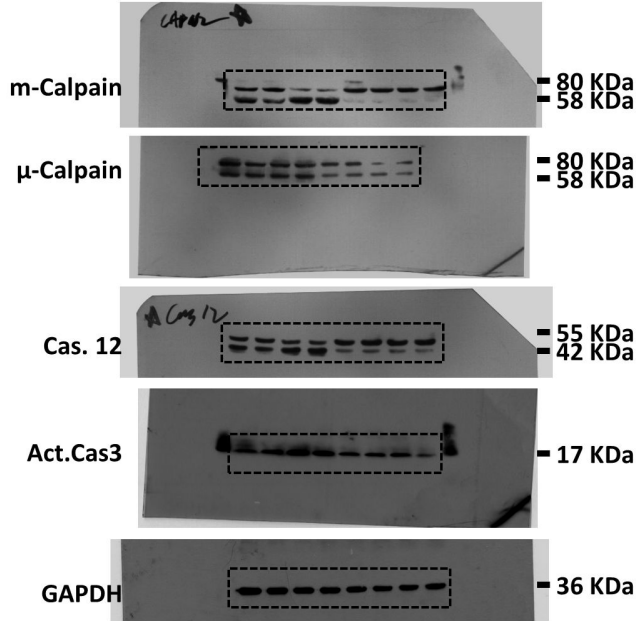

Figure 4E

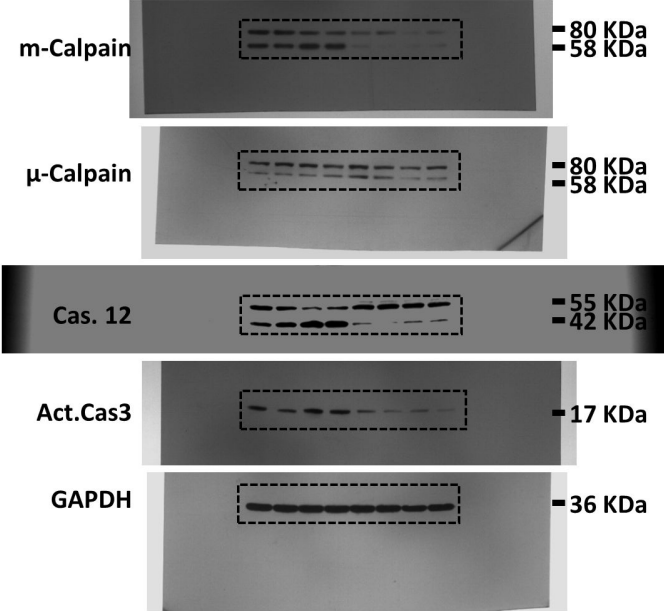

Supplement: Supplementary file 8 — Source Data for Figure 4 [file EMMM-10-e8237-s006.pdf]

Figure 5G

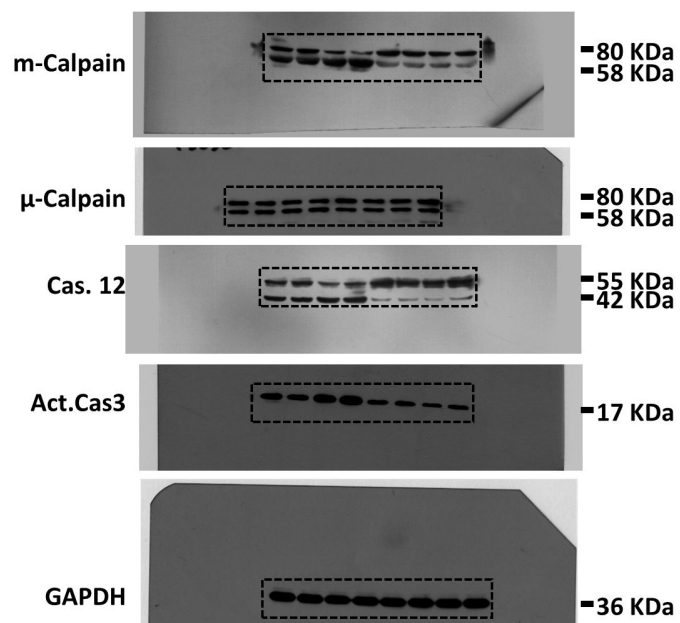

Supplement: Supplementary file 9 — Source Data for Figure 5 [file EMMM-10-e8237-s007.pdf]

Figure 6E

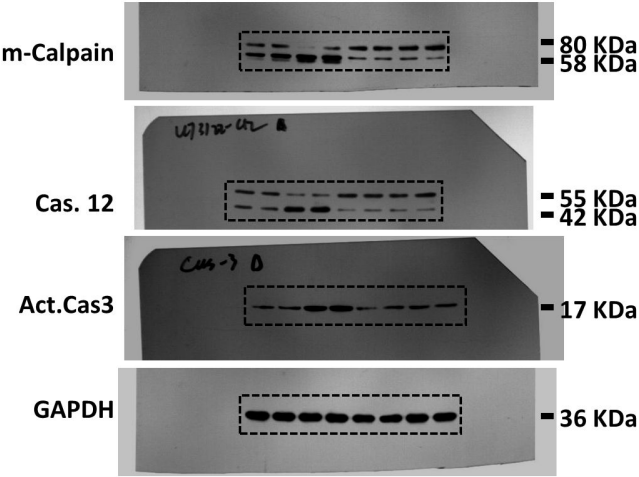

Figure 6F

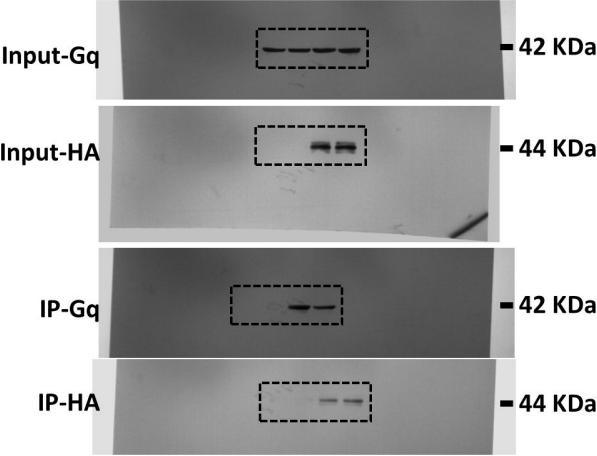

Supplement: Supplementary file 10 — Source Data for Figure 6 [file EMMM-10-e8237-s008.pdf]

Figure 7A

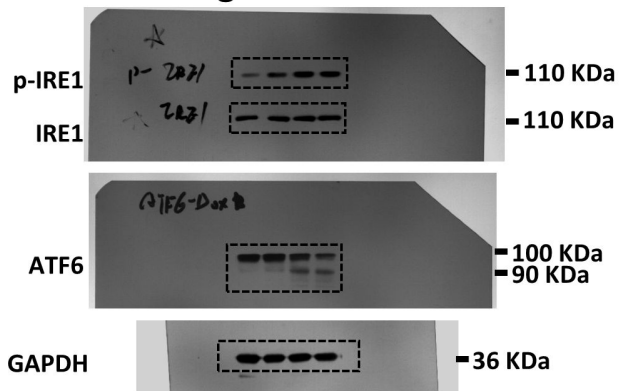

Figure 7I

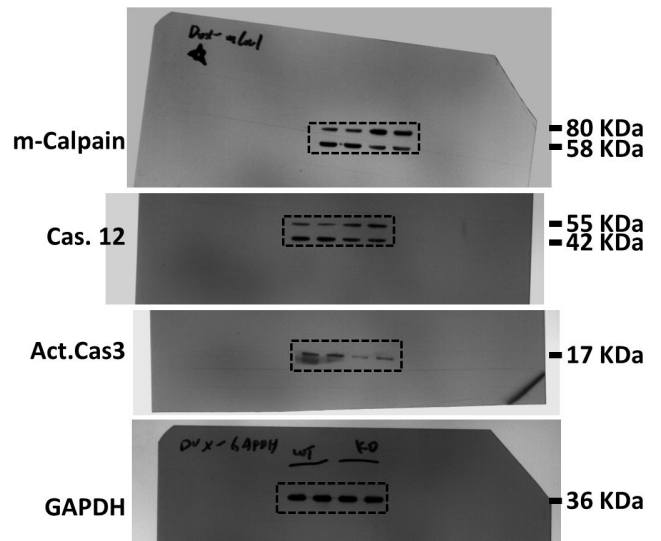

Supplement: Supplementary file 11 — Source Data for Figure 7 [file EMMM-10-e8237-s009.pdf]

Figure 8B

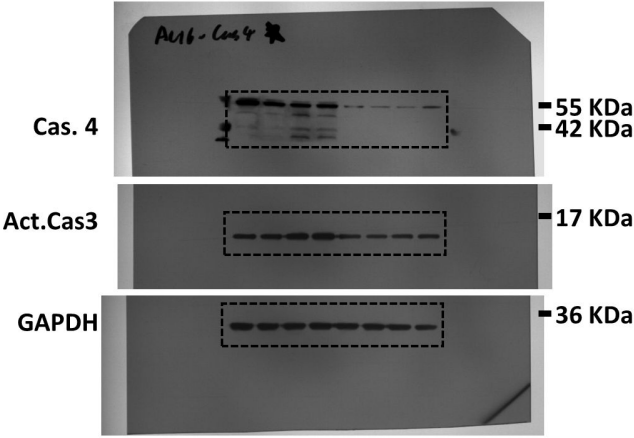

Supplement: Supplementary file 12 — Source Data for Figure 8 [file EMMM-10-e8237-s010.pdf]
